# Temporary Facial Blanching After Botulinum Toxin Injection in Asian: A Case Report and Review of the Literature

**DOI:** 10.1111/jocd.16794

**Published:** 2025-02-07

**Authors:** Hao Chen, Qiuyue Fu, Tianqi Zhang, Gang Chen

**Affiliations:** ^1^ Department of Plastic Surgery Jiangsu Province Hospital of Chinese Medicine Nanjing Jiangsu PR China; ^2^ Nanjing University of Chinese Medicine Nanjing Jiangsu PR China

To the Editor,

Localized facial blanching usually resolves shortly after the botulinum toxin injection. Herein, we report the case of a patient who experienced localized blanching on her forehead and outer canthus following Botulinum toxin type A (BTX‐A) injection for a longer time. This is the first documented occurrence of such a side effect in Asians in the literature. We have conducted a brief analysis of possible reasons for this phenomenon.

We present a case study of a 34‐year‐old Asian female who developed localized blanching on her forehead and outer canthus several hours after receiving BTX‐A (BoNT A, Allergan) injections (Figure 1). The patient received 35U of BTX‐A for forehead wrinkles (C‐line, 14U; four distinct wrinkle areas, 6U) and female pattern hair loss (5 points, 15U) and 6U per side for canthus wrinkles (3 points) at the outer canthus. We performed injection with a (34G, 4 mm) needle. Upon review of the patient's medical history, it was confirmed that she had not received any BTX‐A prior to this incident. The patient notice this change within a few hours of the injection. However, 12 h later, there was still no improvement. The dermatologist conducted a thorough examination and relevant tests (such as blood routine, tests of the coagulation cascade, and Digital Subtraction Angiography), and advised her to continue monitoring and exercising cautiously. The condition was mostly resolved 96 h after the injection. The blanching area near the surrounding skin color did not cause any discomfort to the patient during the procedure.

**FIGURE 1:**
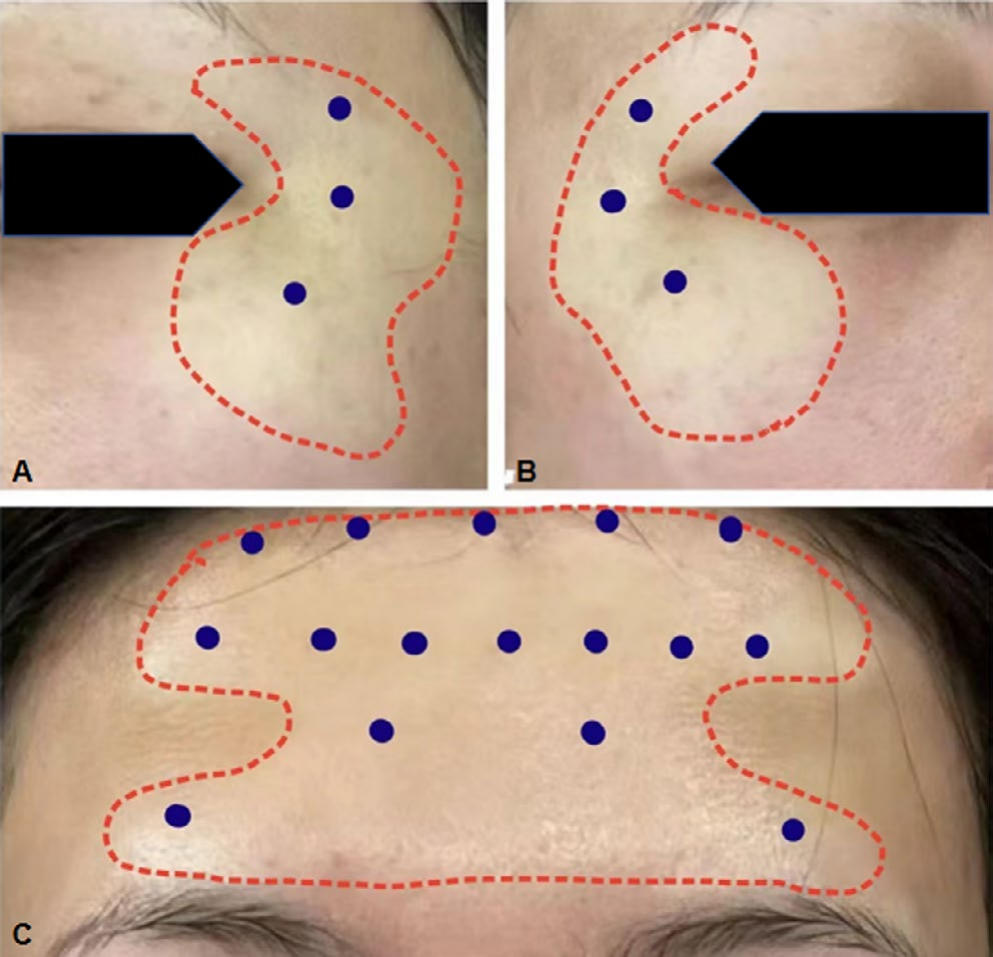
(A)An image of the patient's left side; (B) an image of the patient's right side; (C) a frontal view of the patient. The blue points indicate the injection sites for BTX‐A. The red dashed lines indicate the range and boundaries of the blanching.

We reviewed the factors that may contribute to complications throughout the injection process, from preoperative preparation to postoperative management. These factors include the anesthetic gel, disinfectants, saline, and the postoperative application of ice packs. Subsequently, these potential factors were re‐evaluated in relation to the patient, and no blanching were observed.

The patient denied blanching elsewhere on her body and had not received any additional injections, undergone cosmetic surgery, or experienced facial trauma prior to the onset of symptoms. She is currently not taking any medications and has no family history related to this phenomenon.

## Discussion

We here present a case of a temporary facial blanching after BTX‐A. To the best of our knowledge, this is the first report of this complication which has features of spontaneous remission in Asian. As a rare complication, we aim to elucidate its cause through an examination of the mechanisms of botulinum toxin. Before delving into the mechanism of botulinum toxin, it is essential to have a comprehensive understanding of its function and common complications.

Botulinum toxin injection subcutaneously or intramuscularly to temporarily block nerve impulses between nerves and muscles [1]. This helps weaken muscle strength, reduce facial wrinkles, improve skin elasticity, and shape muscles in a targeted manner. The procedure is simple, quick, with no recovery period, and provides fast results.

Common complications such as drug rash [2], muscle spasm, edema [3], and certain autonomic nervous system symptoms [4], and sarcomatoid granuloma [5], may occur after Botulinum toxin injection. Rare complications include abnormal taste [6] and dizziness [7] following botulinum toxin injection for masseter hypertrophy. A case of myocardial infarction post cystoscopic injection of Botulinum toxin for bladder dysfunction [8] has also been documented. Accidents resulting from botulinum toxin poisoning, overdose, or incorrect placement of injections are not mentioned here.

Khan et al. [9] reported a case of a patient experiencing facial redness and blanching following botulinum toxin injection. Dr. Warren D [10] also documented a similar case. Fouad Mitri et al. [11] reported a case of a 38‐year‐old Caucasian man who had undergone botulinum toxin injections in his forehead and cheeks 3 years prior, presenting with a 2‐year history of facial blanching in these areas.

The observed facial blanching in our patient may be linked to the intricate effects of BTX‐A on neurovascular dynamics. We delve into the pathophysiology of facial blanching post‐BTX‐A injection, focusing on the inhibition of acetylcholine signaling and modulation of the neurovascular network. These mechanisms are interconnected and contribute to the observed clinical features [1, 9]. The localized facial blanching observed in our patient post‐BTX‐A injection can be attributed to several specific mechanisms. First, the decrease in neurovascular components suggests a reduction in blood flow to the blanching areas, aligning with the diminished activity of neurovascular elements due to BTX‐A's influence. BTX‐A likely played a role in stabilizing hyperactive blood vessels within the injection site, which may have directly resulted in the blanching effect [12]. Additionally, the inhibition of the human skin axon reflex and neurogenic vasodilation by BTX‐A offers an explanation for the localized nature of the blanching [6]. Furthermore, by binding to cholinergic nerve terminal glycoprotein structures, BTX‐A potentially blocked acetylcholine secretion, thereby impacting the blood vessels at the injection sites. Lastly, the modulation of the neurovascular network and neuroimmune system by BTX‐A may have contributed to the localized vascular changes noted in our patient's case [13]. It is noteworthy that the effects of BTX‐A are not limited to the muscle tissue but can also influence the surrounding dermal layers due to the diffusion of the toxin and its interaction with the neurovascular units in the skin.

To mitigate the risk of facial blanching, clinicians may consider the following preventive measures: using lower doses of BTX‐A, especially for initial treatments; selecting injection sites away from obvious vascular networks; and ensuring thorough mixing and dilution of the toxin to prevent concentration‐related effects. Additionally, clinicians should obtain a detailed medical history to identify any predisposing factors before administration. For patients experiencing facial blanching, we recommend immediate observation for any progression or associated symptoms. Patients should be advised to avoid pressure or manipulation of the area to prevent exacerbation. It is also important to reassure patients that this condition is typically transient and self‐resolving. We suggest a follow‐up visit within 72 h to assess the condition's resolution and provide additional support or treatment if necessary. Additionally, we underscore the necessity for targeted research to enhance our comprehension of the incidence and underlying mechanisms of vascular complications post‐BTX‐A injections across various ethnicities.

In conclusion, facial blanching as an adverse event post‐BTX‐A injection is rare but significant. Our case contributes to the understanding of BTX‐A complications and suggests the importance of patient education on potential side effects and the need for further research into prevention strategies.

## Conflicts of Interest

The authors declare no conflicts of interest.

## Data Availability

The data that support the findings of this study are available from the corresponding author upon reasonable request.
